# Host MicroRNA hsa-miR-494-3p Promotes EV71 Replication by Directly Targeting PTEN

**DOI:** 10.3389/fcimb.2018.00278

**Published:** 2018-09-03

**Authors:** Qing Zhao, Yuan Xiong, Jingru Xu, Shuang Chen, Pu Li, Yong Huang, Yunying Wang, Wei-Xian Chen, Bo Wang

**Affiliations:** ^1^Department of Laboratory Medicine, The Second Affiliated Hospital of Chongqing Medical University, Chongqing, China; ^2^Department of Laboratory Medicine, Chongqing Health Center for Women and Children, Chongqing, China; ^3^Institute of Microbiology, Chongqing Center for Disease Control and Prevention, Chongqing, China

**Keywords:** EV71, hsa-miR-494-3p, viral replication, apoptosis, PI3K/AKT

## Abstract

Many cellular processes are driven by spatially and temporally regulated microRNAs (miRNAs)-dependent signaling events. Substantial evidence collected over the years indicates that miRNAs are pivotal regulators that contribute to the initiation and development of EV71-related disorders. Importantly, so far, no clinical trial has been undertaken to address the effect of miRNAs on EV71-related diseases. In this study, we show that EV71 infection results in up-regulation of hsa-miR-494-3p levels, and that EV71-induced hsa-miR-494-3p impacts PI3K/Akt signaling pathway by targeting PTEN. However, very little is known about the relationship between hsa-miR-494-3p and EV71 infection. The overall goal of the study is to get a better insight into whether or not hsa-miR-494-3p is involved in the EV71 infection. We found that the EV71 infection induces cellular apoptosis, and that this process can be counteracted by the over-expression of hsa-miR-494-3p mimics. We also present evidence that cell lines deficient in hsa-miR-494-3p are more sensitive to EV71-induced cell death than the corresponding control cells. Collectively, these findings confirm and extend the pervious observation suggesting that disturbances in miRNAs expression can influence EV71 propagation. In addition, they lend strong support to the ideas that hsa-miR-494-3p-mediated signaling pathway plays an important role in the EV71 replication, and that this may have profound implications on our views on EV71-related diseases.

## Introduction

Enterovirus 71 (EV71), a single-stranded positive-sense RNA virus belonging to the *Enterovirus* genus of the *Piconaviridae* family, has been regarded as the second most important neurotropic enterovirus (Ayukekbong and Bergström, 2014). Since EV71 was first identified from an infant suffering from aseptic meningitis in California in 1969, several large epidemic outbreaks have occurred worldwide, particularly in the Asia-Pacific region (Zhu et al., 2014). For example, it has been reported that more than 7 million children suffered from hand, foot, and mouth disease (HFMD) in China between 2008 and 2012, of which 2,457 were fatal. Importantly, about 93% laboratories confirmed that these deaths were associated with EV71 infection (Xing et al., 2014). Currently, the EV71 infection is increasingly viewed as a serious global public health problem, which is associated with diverse clinical consequences including HFMD, herpangina, encephalitis, meningitis, and acute flaccid paralysis (Ooi et al., 2010). Although advances in molecular diagnosis for early diagnosis of EV71 infection have been reported, no effective antiviral agents or specific therapies against EV71 infection have been developed, in part due to the fact that the molecular mechanism of EV71 pathogenesis is poorly understood.

MicroRNAs (miRNAs) are a class of small, highly conserved non-coding RNAs comprising 17-25 nucleotides, which post-transcriptionally suppress the expression of target genes via interacting with target sites located in the 3′ untranslated regions (3′ UTRs) of mRNAs to inhibit their expression (Yates et al., 2013). Since their discovery in 1993, a range of knowledge has been accumulated, showing the importance of miRNA in cellular physiological and pathological processes, including cell development, metabolism, proliferation, differentiation, and apoptosis (Ebert and Sharp, 2012; Yates et al., 2013). Over the past few decades, mounting evidence has supported the notion that the miRNAs may serve as key regulators of viral entry and replication (Wu et al., 2015). This is perhaps best illustrated by the observation that hsa-miR-122, a liver-specific miRNA, is able to bind with the 5′ UTR of hepatitis C virus (HCV) to stabilize HCV genomic RNA (Jopling et al., 2005).

To date, it has become increasingly clear that the miRNAs play a pivotal role in EV71 replication by altering the level of cellular miRNAs, which in turn regulates the expression of viral or host genes (Cui et al., 2010; Wu et al., 2015; Xun et al., 2015). Here, it is noteworthy that the downregulation of DGCR8 (DiGeorge critical region 8), an essential cofactor for the biogenesis of miRNAs, results in the inhibition of EV71 infection (Lui et al., 2014). Remarkably, another research group demonstrated that the EV71-induced up-regulation of hsa-miR-141 expression enhances the switch from cap-dependent to cap-independent translation by targeting the translation initiation factor eIF4E, which increases viral propagation (Ho et al., 2011). Furthermore, the host miRNAs can directly interact with the viral genome to inhibit viral propagation. For example, it has been demonstrated that (i) miR-296-5p significantly inhibits EV71 replication by interacting with nt (nucleotides) 2,115–2,135 and nt 2,896–2,920 of the viral genome (Zheng et al., 2013); and (ii) the upregulation of miR-23b suppresses the replication of EV71 by directly targeting at EV71 VP1 (Wen et al., 2013). In addition, the host miRNA may affect EV71 replication by regulating the immune signaling pathways. In this context, it is informative to mention that (i) hsa-miR-526a positively regulates type I interferon production to inhibit EV71 replication by targeting the CYLD (cylindromatosis) to enhancement of RIG-I K63-linked ubiquitination (Xu C. et al., 2014); (ii) hsa-miR-548 suppresses the host antiviral responses by directly targeting IFN-λ1 (interferon-λ1), which in turn promotes EV71 infection (Li et al., 2013); and (iii) the upregulation of hsa-miR-146a induced by EV71 targets IRAK1 (Interleukin-1 receptor-associated kinase 1) and TRAF6 (tumor necrosis factor-associated factor), both of which are associated with TLR (toll-like receptor) signaling pathway and type I interferon production (Ho et al., 2014). Interestingly, although hsa-miR-1246 does not influence the EV71 infection, it plays an important role in EV71-related neurological pathogenesis by targeting *DLG* (disc-large homolog 3) gene, which is involved in neurological disorders (Xu L. J. et al., 2014). However, so far, no clinical trial has been conducted to our knowledge relating to therapeutic candidate miRNA treating EV71-mediated diseases. In order to find the potent therapeutic miRNA for treating EV71-related disorders, more research also needs to be done to investigate the molecular mechanisms underlying the crosstalk between miRNAs and EV71 infection.

In this study, we found that (i) EV71 infection can induce the up-regulation of expression of hsa-miR-494-3p, which promotes viral propagation; (ii) the PI3K/Akt signaling pathway is activated by targeting PTEN via hsa-miR-494-3p in EV71 infected cells; and (iii) activating PI3K/Akt signaling pathway can suppress the early apoptosis induced by EV71 infection, which in turn facilitates an appropriate environment for viral replication.

## Materials and methods

### Cell culture and transfection

Rhabdomyosarcoma (RD) and human embryonic kidney (HEK) 293 cells were obtained from American Type Culture Collection (ATCC, USA). Unless specified otherwise, all cells were cultured at 37°C in a humidified 5% CO_2_ incubator in minimum essential medium (MEM, Gibco) supplemented with 2 or 10% (v/v) heat-inactivated fetal bovine serum (FBS, Gibco). RD cells or HEK293 cells were transiently transfected with miR-494 mimics (MC12409), miR-494 inhibitors (MH12409), miRNA negative control (miR-NC), mutant PTEN siRNA (CST, #6201), and PTEN siRNA (CST, #6538) using Lipofectamine 2000 (Invitrogen). The miRNAs and siRNAs were commercially purchased by Thermo Fisher and Cell Signaling, respectively.

### Virus infection and titration

EV71 strain was a kind gift from Ms. Xu, Chongqing Centers for Disease Control and Prevention, and propagated on RD cells or HEK 293 cells. Briefly, the confluent cells were infected with the wild type EV71 at a multiplicity of infection (MOI) 1 in MEM containing 2% FBS. Next, virus stocks were collected from supernatants at day 3 post-infection and stored at 80°C. EV71 virus titers were determined by the 50% tissue culture infectious dose (TCID50) assay using RD cells or HEK 293 cells as previously mentioned (Wang et al., 2012). Note that the Reed-Münch endpoint calculation method was employed to determine the virus titer. At least three independent experiments were carried out for each condition.

### PI3K inhibitor

The PI3K inhibitor Wortmannin (Sigma) was freshly dissolved at 20 mM in DMSO, then added to a medium at a final concentration of 100 nM for 1 h prior to virus infection, and kept in the medium throughout the experiment, or added at different intervals as described in the text or figure legends.

### Antibodies

The mouse polyclonal antibody against EV71 VP1 (~36 kDa, Abcam), the mouse monoclonal antibodies against PTEN (~54 kDa, Cell Signaling) and β-actin (~42 kDa, Abcam), the rabbit monoclonal antibodies against Akt (~60 kDa, Cell Signaling) and phosphor-Akt (~60 kDa, Cell Signaling), and the goat anti-mouse and anti-rabbit IgGs conjugated to horseradish peroxidase (HRP). In addition, the mouse polyclonal antibody against EV71 VP1 (Abcam) and the goat anti-mouse Alexa Fluor 594 secondary antibody (Invitrogen) were used for the immunofluorescence assay in this study.

### RNA extraction and real time quantitative PCR

Viral RNA and miRNA were prepared using the Trizol reagent (Invitrogen) according to the manufacturer's instructions. Here, it should be mentioned that, under our condition, we don't employ 70% ethanol to wash miRNA pellet due to the fact that 70% ethanol can disrupt small RNA profiles. Then, 1 μg of total RNA was reverse transcribed into cDNA with the PrimeScript RT reagent kit (Takara). For hsa-miR-494-3p expression detection, real time quantitative PCR (RT-PCR) was performed using a TaqMan miRNA assay kit (002365, Thermo Fisher) according to the manufacturer's instructions. For the viral RT-PCR, a quantitation standard curve was achieved using seven 10-fold serial dilutions of the recombinant plasmid standard DNA, which contained a 226 bp sequence derived from the VP1 gene of EV71, varying from 1 × 10^3^ to 1 × 10^9^ copies. Real-time quantitative (Q-PCR) was performed by targeting the VP1 gene in 96-well plates with CFX Connect^TM^ real time PCR detection system (Bio-Rad, California, USA). Each 20 μl of reaction contained 10 μl of 2× SYBR Green Supermix, 20 nM of target gene primer mix, and 20–50 ng of a cDNA template. The sequence of the primers used was as follows: Forward: 5′-GCA GCC CAA AAG AAC TTC AC-3′ and Reverse: 5′-ATT TCAGCA GCT TGG AGT GC-3′. The PCR reaction was set up as follows: initial denaturation step at 95°C for 10 min, followed by 40 cycles of 30 s at 94°C, at 55°C for 30 s and at 72°C for 30 s. Quantified results for the experimental samples were extrapolated from the standard curve, with all experimental samples being run in triplicate.

### Western blot

Western blots were processed as previously described (Wang et al., 2013). Briefly, cells were lysed in a buffer containing 20 mM Hepes (pH 7.4), 100 mM NaCl, 5 mM EDTA (pH 7.4), 1 mM Na_3_VO_4_, 30 mM NaF, 5% glycerol, 0.1% SDS, 1% Triton X-100, 10 mM p-nitrophenylphosphate, and 1 mM glycerophosphate, supplemented with complete protease inhibitors (Selleck). Cell lysates were obtained by centrifugation at 13,000 rpm and 4°C, and the total protein concentration was determined by the Bicinchoninic Acid Protein Assay Kit (Pierce). The proteins were resolved by sodium dodecyl sulfate polyacrylamide gel electrophoresis (SDS-PAGE) and transferred to PVDF membranes (Millipore). The membranes were blocked for 2 h with 5% non-fat dry milk solution in Tris-buffered saline containing 0.1% Tween 20. The membranes were then blotted with specific primary antibodies, followed by incubation with secondary antibodies conjugated with horseradish peroxidase. The blots were developed with an enhanced chemiluminescent substrate (ECL) or SuperSignal West Femto Maximum Sensitivity Substrate (Pierce).

### Assessment of cell viability

Cell viability was evaluated by using the 3-(4,5-dimethylthiazol-2-yl)-2,5-diphenyltetrazolium bromide (MTT) assay. Briefly, ±5 × 10^3^ cells/well were seeded into a 96-well culture plate (8 wells per condition; 200 μl medium per well) and cultivated for 1 day under standard conditions (see above). Next, the cells were treated with hsa-miR-494 mimics, hsa-miR-494 inhibitors, corresponding negative control (NC) or Wortmannin (100 nM), and cultivated for 72 h. Then, 20 μl MTT (5 mg/ml in PBS) was added to each well, and the cells were re-incubated at 37°C to allow the viable cells to produce formazan. After 4 h, the medium was replaced by 150 μl DMSO and the cell plate was vibrated for 10 min at room temperature to dissolve the formazan precipitates. The absorbance of the colored solution was measured at 570 nm by using a Multiskan™ FC microplate reader (Thermo Scientific, MA USA). A reference wavelength of 690 nm was used to correct for any turbidity in the samples. At least three independent experiments were performed for each condition.

### Immunofluorescence assay

RD cells or HEK 293 cells were seeded on coverslips. To visualize caspase-3 activity in real time, the cells were incubated with 5 μM NucView 488 (Biotium) for 30 min prior to virus infection and kept in the medium throughout the experiment, or added at different intervals as described in the text or figure legends. The cells were then washed three times with ice-cold PBS and fixed with 4% paraformaldehyde for 20 min at room temperature. Next, the cells were permeabilized with 0.25% Triton X-100 for 10 min, and blocked with goat serum for 1 h. Finally, the cells were incubated with diluted mouse anti-EV71 antibody followed by goat anti-mouse Alexa 594 secondary antibody (red). The cell images were observed using an Olympus IX 73 inverted fluorescence microscope with cellSens imaging software. The images shown are representative of three independent experiments.

### Luciferase assay

The 3′-UTR of PTEN was amplified by PCR using genomic DNA and cloned into the *NheI* and *XhoI* sites in the pGL3 vector (Promega). The mutant 3′-UTR of PTEN was also generated by PCR and cloned into the *NheI* and *XhoI* sites in the pGL3 vector (Promega). RD cells or HEK293 cells were seeded in 96 well plates and transiently transfected with luciferase reporter plasmids (wt-PTEN-UTR-pGL3 or mt-PTEN-UTR-pGL3), the control plasmid pRL-TK-*Renilla* vector (Promega), and hsa-miR-494-3p mimics or negative control. After 48 h, the cells were collected and the luciferase activities were measured using the dual-luciferase reporter assay system, according to the manufacturer's instructions (Promega).

### Flow cytometry

The percentage of apoptotic cell was measured by Nucview 488 (Biotium) according to the manufacturer's protocol (Cen et al., 2008). Briefly, the RD cells or HEK 293 cells were transfected with hsa-miR-494-3p mimics, hsa-miR-494-3p inhibitors, negative control, or cultured with PI3K/Akt inhibitor Wortmannin. The cells were then infected with or without EV71. At indicated time points post-infection, the cells were collected and washed with ice-cold PBS, and incubated with 5 μM NucView 488 (Biotium) at room temperature in the dark for 30 min. For each experiment, 2 × 10^5^ cells were analyzed on flow cytometer (BD). At least three independent experiments were performed for each condition.

### Statistical analysis

Statistics were performed on the VassarStats statistical computation website (http://vassarstats.net/). One-way analysis of variance was used to determine the differences among the independent groups of numerical values, and the individual differences were further explored with a Student's *t-*test. Three significance levels were chosen: *p* > 0.05 (no significant difference; NS), *p* < 0.05 (medium significance), and *p* < 0.01 (high significance).

## Results

### The endogenous hsa-miR-494-3p level is up-regulated in response to EV71 infection

**Figure 1:**
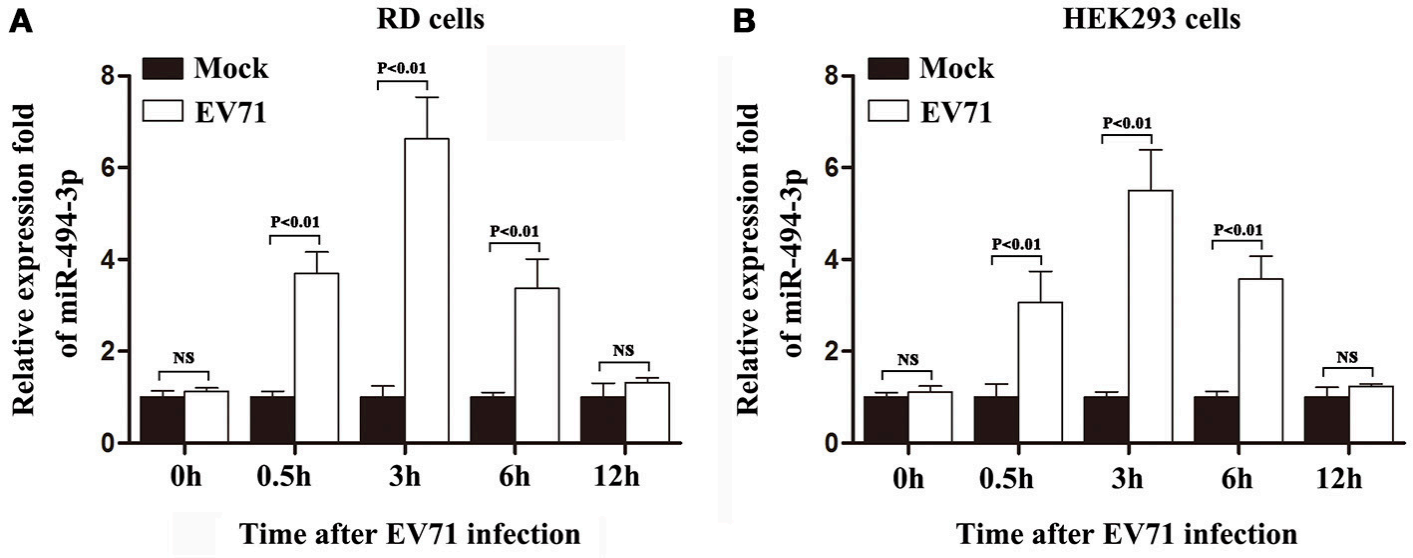
EV71 infection up-regulated expression of hsa-miR-494-3p in RD and HEK 293 cells. The cells were exposed (open bars) or not exposed (closed bars) to EV71 infection at an MOI of 1. The expression of hsa-miR-494-3p was quantified using qRCR at indicated time points after EV71 infection. U6 was used as an internal control. The results represent the mean ± standard deviation of three independent experiments and present the relative expression levels of hsa-miR-494-3p in RD **(A)** and HEK 293 **(B)** cells (*p* < 0.01 by ANOVA).

Previously, by employing microarray assay and quantitative RT-PCR, it has been reported that the expression of hsa-miR-494-3p is up-regulated in EV71-infected RD cells (Xun et al., 2015). Additionally, the results of one resent study suggest that, in patients with EV71 mild symptom, the serum level of hsa-miR-494-3p is induced by viral infection (Wang et al., 2016). However, to date, the pathophysiological relevance of this observation remains to be established. To further our understanding of the roles of hsa-miR-494-3p in EV71 infection, we first validated the expression levels of intracellular hsa-miR-494-3p in EV71-infected RD and HEK 293 cells. The growth arrested RD and HEK 293 cells were infected with EV71 at an MOI of 1. The cells were collected at different time points post infection (pi) and the expression levels of hsa-miR-494-3p were measured by RT-PCR. The results of these validation experiments demonstrated that the expression hsa-miR-494-3p was up-regulated in response to EV71 infection in our RD and HEK 293 cell culture systems, and that the infection of cells by EV71 induced the level of hsa-miR-494-3p with a transient peak at 3 hpi (Figure 1). Therefore, the intracellular hsa-miR-494-3p expression was significantly increased in these infected two cell lines. Here, it should be noted that Ultraviolet (UV)-irradiated EV71 only triggers the upregulated expression of hsa-miR-494-3p at 30 min pi (Supplementary Figure 1).

### The over-expression of hsa-miR-494-3p promotes EV71 replication

**Figure 2:**
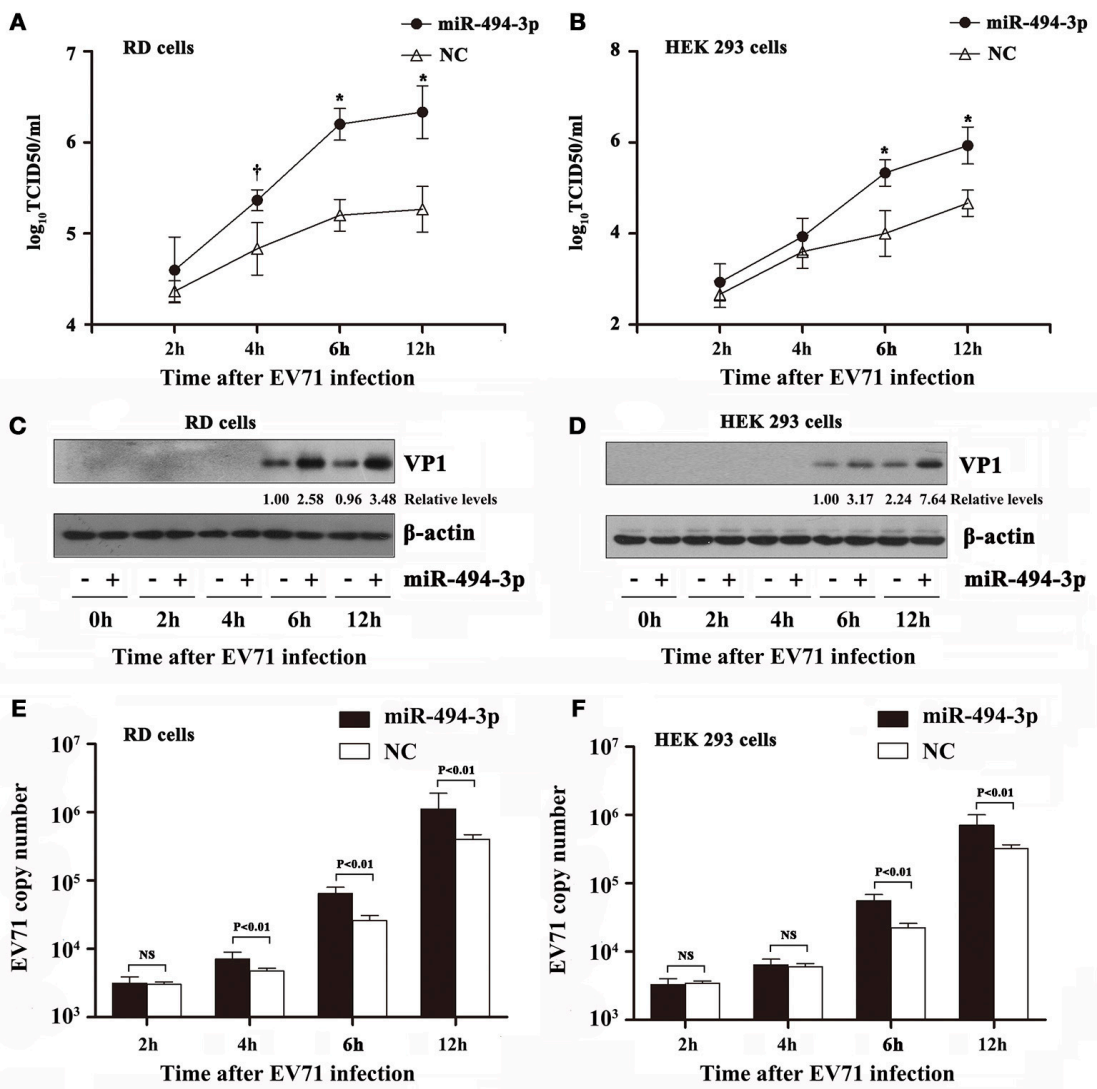
Effects of over-expression of hsa-miR-494-3p on EV71 infection. RD and HEK 293 cells were transfected with hsa-miR-494-3p mimics or the negative control (NC) for 48 h, and then followed by EV71 infection for 2, 4, 6, 12 h at an MOI of 2. Virus titers in culture supernatants from RD **(A)** and HEK 293 **(B)** were measured at the indicated time post-infection. The cells were collected at different time points as indicated. RD **(C)** and HEK 293 **(D)** cell lysates were blotted with anti-EV71 VP1 and anti-β actin antibodies. β actin was used as an internal control. Next, qRT-PCR was employed to determine the EV71 VP1 mRNA levels in RD **(E)** and HEK 293 **(F)** cells. The data shown are the mean ± standard deviation. The experiments were repeated three times (†*P* < 0.05, ^*^p < 0.01 by ANOVA).

Since the expression level of hsa-miR-494-3p is up-regulated in EV71-infected cells, it is intriguing to investigate whether the over-expression of hsa-miR-494-3p affects EV71 replication. To elucidate the effect of hsa-miR-494-3p on EV71 replication, the RD and HEK 293 cells were transfected with hsa-miR-494-3p *mir*Vana mimics, followed by infection with EV71 at an MOI of 2. The titers (TCID50) of EV71 were determined at different times post-infection. As shown in Figure 2A, the EV71 titers in RD cells with over-expression of hsa-miR-494-3p were significantly higher than those of the negative control at 4, 6, and 12 hpi. In HEK 293 cells, over-expression of hsa-miR-494-3p significantly increased the EV71 titers at 6 and 12 hpi compared to those of the cells treated with the negative control (Figure 2B). These observations indicated that the hsa-miR-494-3p considerably promote EV71 replication.

To further confirm and extend these previous observations that hsa-miR-494-3p could enhance EV71 propagation, western blotting and qRT-PCR were employed to determine the protein abundance and viral mRNA levels, respectively. Western blotting results demonstrated that the EV71 VP1 protein levels were markedly increased in the hsa-miR-494-3p transfected cells at 6 and 12 hpi in RD and HEK 293 cells (Figures 2C,D). Finally, a real time PCR assay was conducted to quantify the EV71 VP1 gene. These data revealed that the VP1 RNA at 4, 6, and 12 hpi were significantly increased in EV71-infected RD cells pre-transfected with hsa-miR-494-3p (*p* < 0.01; Figure 2E). Additionally, compared to the negative control group, the EV71 VP1 RNA levels in the hsa-miR-494-3p-tranfected HEK 293 cells were significantly increased at 6 and 12 hpi (*p* < 0.01; Figure 2F). Taken together, our findings suggest that hsa-miR-494-3p plays an important role in the amplification of EV71.

### Silencing of hsa-miR-494-3p inhibits EV71 propagation

**Figure 3:**
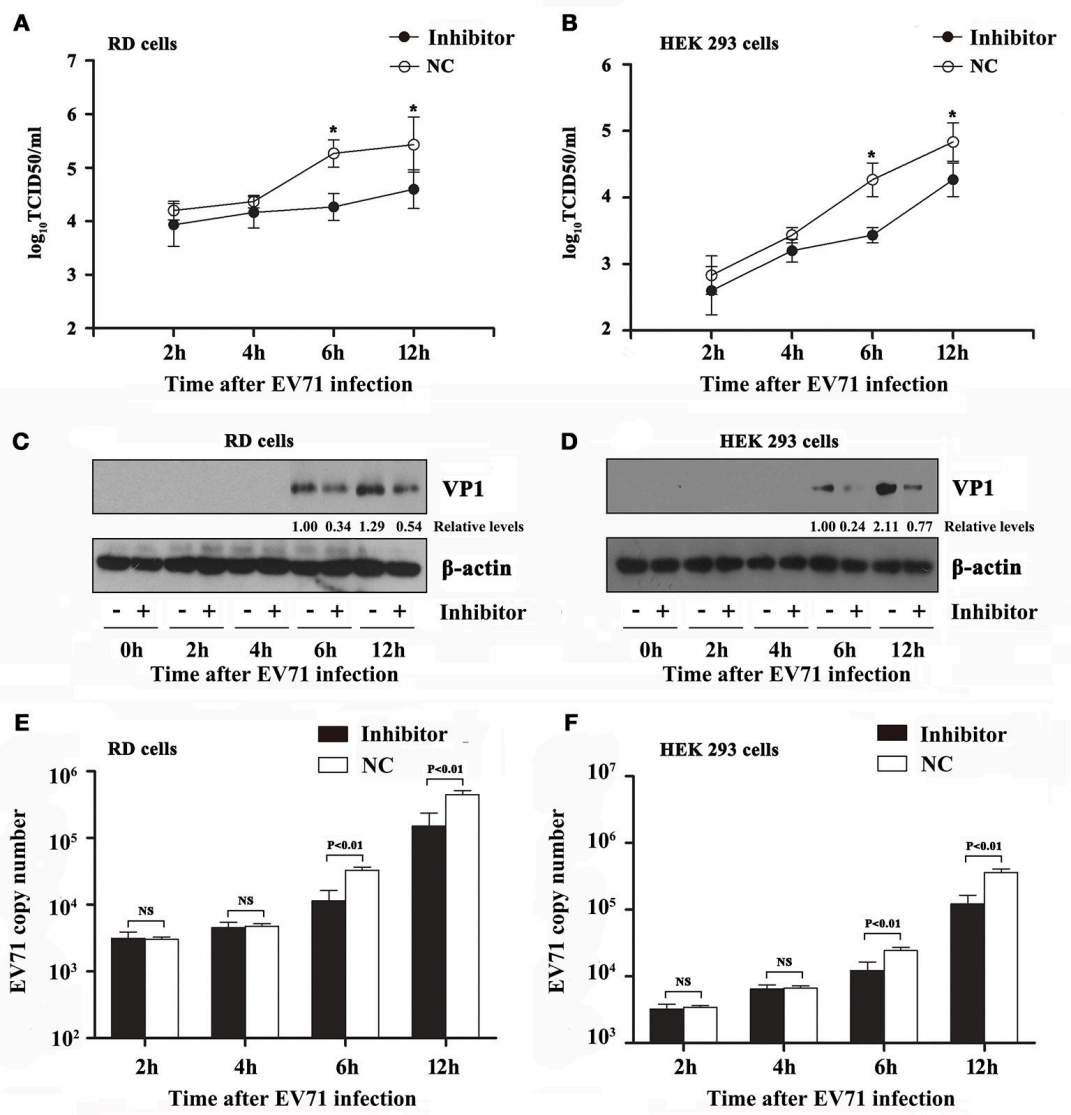
Effects of hsa-miR-494-3p inhibitors on EV71 infection. RD and HEK 293 cells were transfected with hsa-miR-494-3p inhibitors or the negative control (NC) for 48 h, and then followed by EV71 infection for 2, 4, 6, 12 h at an MOI of 2. Virus titers in culture supernatants from RD **(A)** and HEK 293 **(B)** were measured at the indicated time post-infection. The cells were collected at different time points as indicated. RD **(C)** and HEK 293 **(D)** cell lysates were blotted with anti-EV71 VP1 and anti-β actin antibodies. β actin was used as an internal control. Next, qRT-PCR was employed to determine the EV71 VP1 mRNA levels in RD **(E)** and HEK 293 **(F)** cells. The data shown are the mean ± standard deviation. The experiments were repeated three times (^*^p < 0.01 by ANOVA).

To strengthen our observation that hsa-miR-494-3p promotes EV71 infection, we also investigated whether the cells lacking hsa-miR-494-3p are more resistant to EV71 replication. The RD and HEK 293 cells were transfected with the hsa-miR-494-3p inhibitor, a chemically modified single-stranded oligonucleotides that irreversibly bind and inactivate the endogenous hsa-miR-494-3p. Consistent with the positive effect of hsa-miR-494-3p on EV71 replication, the EV71 titers at 6 and 12 hpi were significantly suppressed by the hsa-miR-494-3p inhibitors compared to the negative control in both cell lines (*p* < 0.01; Figures 3A,B). Next, western blotting was applied to detect the EV71 VP1 protein. The blotting data also revealed that the EV71 VP1 protein levels were evidently reduced in RD-infected cells and HEK 293-infected cells at 6 and 12 hpi (Figures 3C,D). Finally, the level of EV71 RNA in cells transfected with the hsa-miR-494-3p inhibitor was significantly lower than that of the negative-control group at 6 and 12 hpi (Figures 3E,F). In RD-infected cells, the EV71 VP1 RNA levels in the negative control group were 2.9- and 3.0-folds higher than those of the inhibitor-transfected group at 6 and 12 hpi, respectively (Figure 3E). Furthermore, the RT-PCR results in the HEK 293 cells revealed that the hsa-miR-494-3p inhibitors significantly decreased the EV71 VP1 RNA levels by 50 and 66% at 6 and 12 hpi, respectively (*p* < 0.01; Figure 3F). Collectively, these results indicate that the inhibition of hsa-miR-494-3p may suppress EV71 infection.

### The hsa-miR-494-3p targets 3′UTR of PTEN and affects the PI3K/AKT signaling pathway

**Figure 4:**
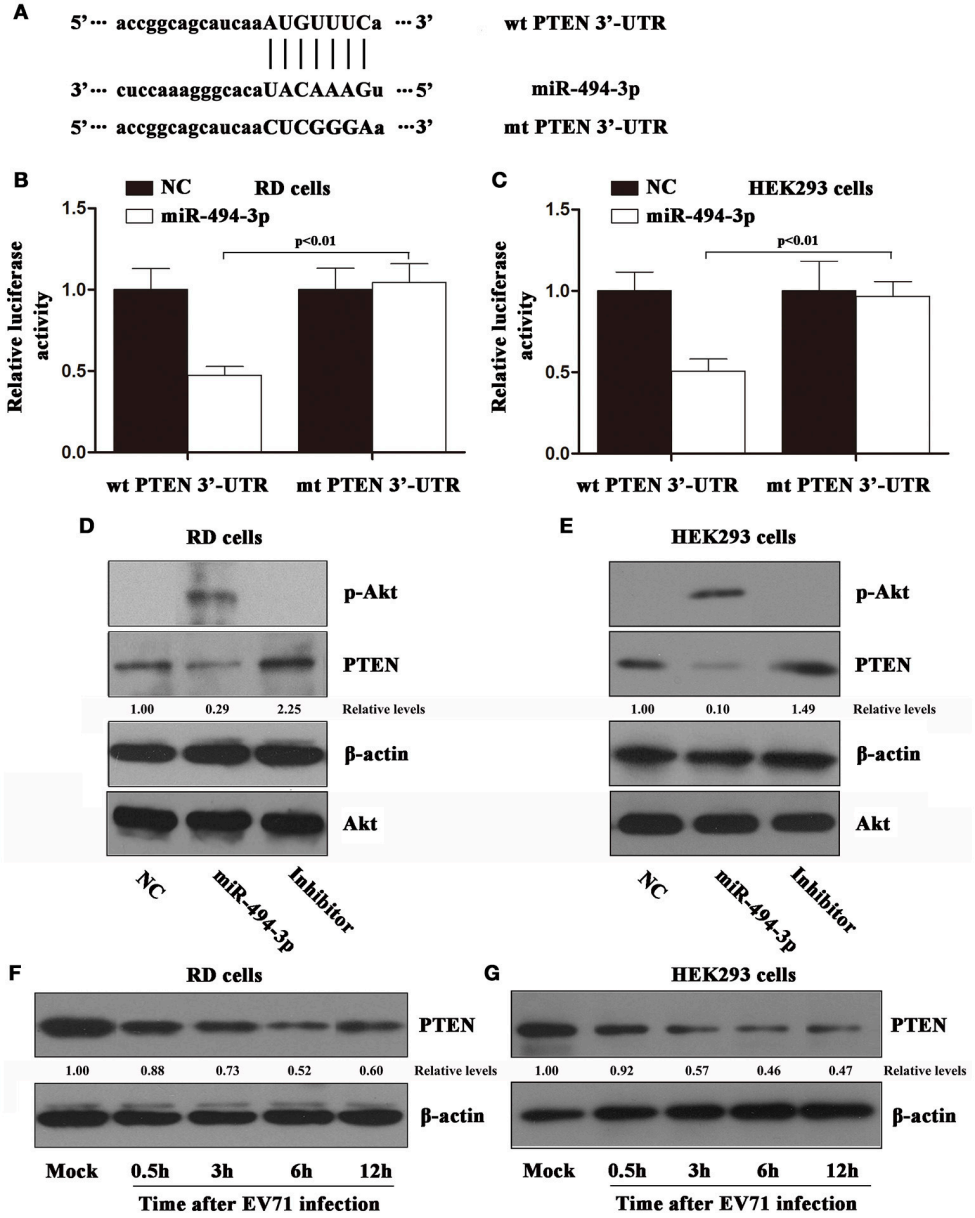
PTEN is a target of EV71-induced hsa-miR-494-3p. **(A)** The putative hsa-miR-494-3p binding site within the PTEN 3′ UTR is predicted by TargetScan, and the mutation form is indicated. RD and HEK 293 cells were transiently co-transfected with luciferase reporter plasmids containing the hsa-miR-494-3p target sites (wt PTEN 3′UTR) or corresponding mutants (mt PTEN 3′UTR) and hsa-miR-494-3p mimics, or negative control, and cultured in a standard growth medium. After 24 h, luciferase reporter assays were performed in RD **(B)** and HEK 293 cells **(C)**. The data shown are the mean ± standard deviation. The cells were transiently transfected with hsa-miR-494-3p mimics, inhibitors, or negative control. After 24 h, the RD **(D)** and HEK 293 **(E)** cells were collected, and then the lysates were blotted with anti-phospho Akt, anti-PTEN, anti-Akt, and anti-β actin antibodies. The cells were exposed to EV71 infection for 0.5, 3, 6, 12 h at an MOI of 2. The RD **(F)** and HEK 293 **(G)** cell lysates were blotted with anti-PTEN and anti-β actin antibodies. β actin was used as an internal control. The experiments were repeated three times (*p* < 0.01 by ANOVA).

To gain a better understanding of how hsa-miR-494-3p influences the EV71 infection, we employed the web-based miRNA target prediction database TargetScan (http://www.targetscan.org/) and MicroRNA.org (http://www.mirbase.org/) to identify the potential hsa-miR-494-3p targets. Here, we found that hsa-miR-494-3p has the seed region that matches the 3′UTR of PTEN (nucleotides 2,784–2,790, Figure 4A). Additionally, over the past few decades, accumulating evidence supports the notion that PTEN is a direct target of hsa-miR-494-3p in various types of cancer (Li et al., 2015; Liu et al., 2015). However, the relationship between PTEN and miR-494 in EV71 infection remains elusive. To verify the possibility that PTEN is regulated by hsa-miR-494-3p in EV71 infection, we cloned the luciferase reporter vectors containing either wild-type or mutant PTEN 3′UTR sequence. The relative luciferase activities were measured after co-transfecting with either hsa-miR-494-3p mimics or negative control. We observed that the hsa-miR-494-3p mimics dramatically decreased the luciferase activities of wild type PTEN 3′UTR in RD and HEK 293 cells compared with cells transfected with the negative control (Figures 4B,C). In contrast, the mutation in PTEN 3′UTR completely abolished the effect of hsa-miR-494-3p to reduce luciferase activity in both cell lines (Figures 4B,C). Next, we examined whether the hsa-miR-494-3p expression could inhibit the endogenous PTEN expression. Therefore, we assayed cells with over-expression of hsa-miR-494-3p mimics, inhibitors, or the negative control. These findings suggest that the over-expression of hsa-miR-494-3p levels significantly suppress the PTEN expression in RD and HEK 293 cells, while inhibition of hsa-miR-494-3p expression obviously increase the PTEN expression in both cell lines (Figures 4D,E). Here, it is noteworthy that the PTEN expression was inhibited by hsa-miR-494-3p, which in turn resulted in the activation of the PI3K/Akt signaling pathway (Figures 4D,E). To further substantiate the interpretation of these observations, we also investigated whether the PTEN expression could be impacted by EV71 infection. The results of these experiments showed that the EV71 infection could affect the PTEN expression in RD and HEK 293 cells (Figures 4F,G). Importantly, the inhibition of hsa-miR-494-3p expression can abolish EV71-mediated suppression of PTEN (Supplementary Figure 2). Furthermore, downregulation of PTEN significantly enhances EV71 replication (Supplementary Figure 3).

### The PI3K/AKT signaling pathway is necessary and sufficient for EV71 replication

**Figure 5:**
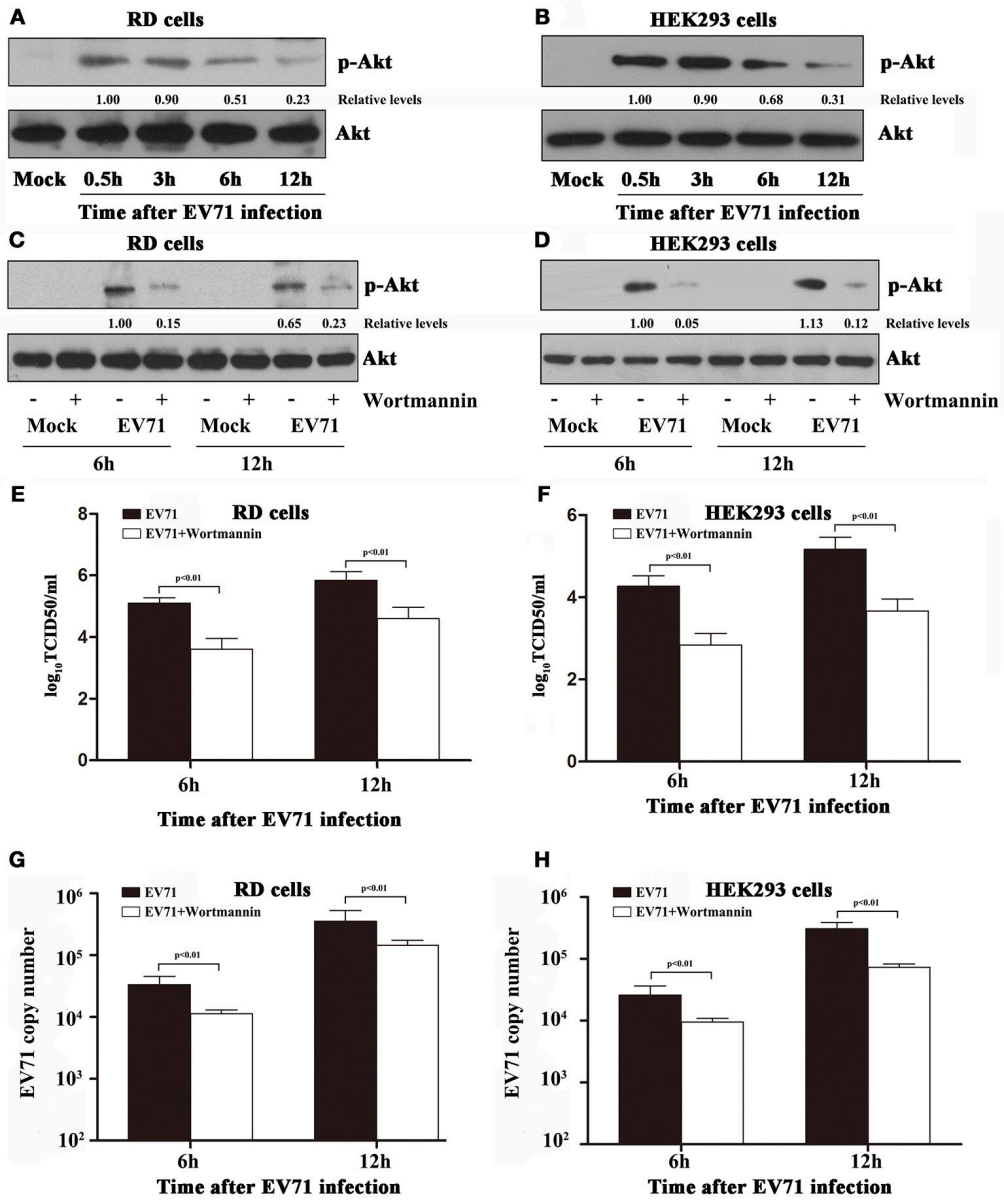
Infection with EV71 resulted in the activation of PI3K/Akt signaling pathway in RD and HEK 293 cells. **(A,B)** RD and HEK293 cells were infected with EV71 at an MOI of 2. At the indicated time points post-infection, the cells were collected and lysed for blotting analysis to detect phospho Akt (p-Akt) and β actin. **(C,D)** RD and HEK293 cells were pre-incubated without or with Wortmannin (100 nM) for 1 h, and then followed by infection with EV71 at an MOI of 2. Wortmannin was present throughout the experiments. The cells and supernatants were collected separately at different time points as indicated. Cell lysates were blotted with anti-phospho Akt and anti-β actin antibodies. β actin was used as an internal control. **(E,F)** Virus titers in the supernatants collected in panels **(C,D)** were measured by TCID_50_. **(G,H)** qRT-PCR was employed to determine the EV71 VP1 mRNA levels in RD and HEK 293 cells. The data shown are the mean ± standard deviation. The experiments were repeated three times (*p* < 0.01 by ANOVA).

Activation of the PI3K/Akt signaling pathway has previously been shown to play a key role in EV71 infection (Wong et al., 2005; Zhang H. et al., 2014). As mentioned before, the PI3K/Akt signaling pathway was activated by hsa-miR-494-3p up-regulation (Figures 4D,E). To gain better insight into the role of hsa-miR-494-3p-mediated Akt signaling pathway in EV71 replication, we first investigated the kinetics of EV71 induced-activation of the Akt signaling pathway. We found that the infection of RD cells by EV71 induced Akt phosphorylation with a transient peak at 0.5 hpi and sustaining at a high level until for at least 12 h compared to the mock group (Figure 5A). A similar activation of the Akt signaling pathway was also observed in EV71-infected HEK 293 cells (Figure 5B). Given the fact that the hsa-miR-494-3p mimics or inhibitors could significantly influence EV71 replication at 6 and 12 hpi compared to the corresponding control, the cell samples and supernatants were collected at 6 and 12 hpi with EV71 infection in all of the subsequent experiments. To further examine whether the EV71 infection promotes Akt activation via its upstream target PI3K, the RD and HEK 293 cells were treated with Wortmannin, a potent and specific inhibitor of PI3K. The results showed that Wortmannin effectively inhibited the EV71-induced Akt phosphorylation in both cell lines (Figures 5C,D). Furthermore, viral titers and RT-PCR were employed to determine the effect of the hsa-miR-494-3p-mediated Akt signaling pathway on EV71 replication. We observed that (i) the EV71 titers were significantly reduced by PI3K inhibitor at 6 and 12 hpi compared to the mock group in RD and HEK 293 cells (Figures 5E,F), and (ii) the level of EV71 RNA in the cells treated with the Wortmannin was significantly lower than that of the negative-control group at 6 and 12 hpi (Figures 5G,H).

### The hsa-miR-494-3p exhibits inhibitory effect on EV71-induced apoptosis

**Figure 6:**
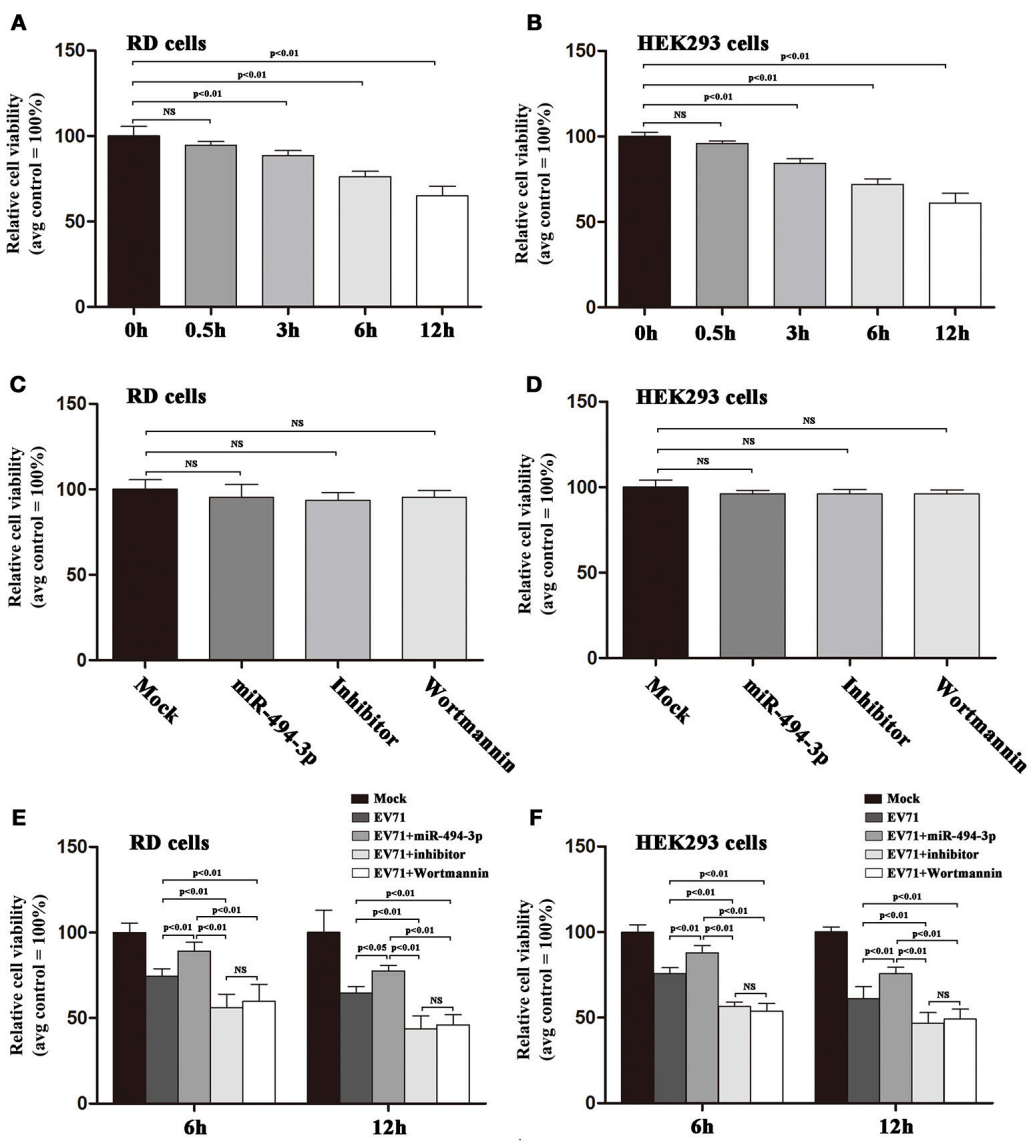
Upregulation of hsa-miR-494-3p ameliorates cell death induced by EV71 infection. **(A,B)** RD and HEK293 cells were infected with EV71 at an MOI of 2. At the indicated time points post-infection, MTT assay was employed to evaluate the cell viability. **(C,D)** RD and HEK293 cells were pre-incubated with Wortmannin (100 nM), or transfected with hsa-miR-494-3p mimics or inhibitors for 48 h. Cell viability was evaluated by MTT assay, and expressed as the percentage of the average value of the corresponding non-treated condition. **(E,F)** RD and HEK293 cells were transfected with or without hsa-miR-494-3p mimics or inhibitor for 48 h, or pre-incubated with or without Wortmannin (100 nM) for 1 h, and then followed by infection with EV71 for 6 and 12 h at an MOI of 2. MTT assay was employed to evaluate the cell viability at the indicated time points. The experiments were repeated three times (*p* < 0.05, *p* < 0.01 by ANOVA).

Currently, it is widely accepted that the activation of PI3K/Akt signaling pathway could initiate survival signals in response to diverse stimuli in many different cell lines (Manning and Toker, 2017). Additionally, our pervious findings demonstrated that the EV71 infection could lead to cell death (Wang et al., 2012). In this study, we confirm these findings by showing that EV71 infection could induce cell death in RD and HEK 293 cells (Figures 6A,B). To gain a better insight into the potential mechanism underlying the increased EV71 replication via hsa-miR-494-3p, we investigated the role of hsa-miR-494-3p-mediated PI3K/Akt in regulating the survival signals in EV71 infected cells. These experiments revealed that (i) the cells with over-expression of hsa-miR-494-3p significantly abrogated EV71-induced cell death, (ii) the cells lacking hsa-miR-494-3p or incubating Wortmannin are more sensitive toward EV71-induced cell death than the corresponding control cells (Figures 6C–F), and (iii) UV-irradiated EV71 infection with hsa-miR-494-3p mimic, inhibitor, or Wortmannin cannot induce cell death (Supplementary Figure 4).

**Figure 7:**
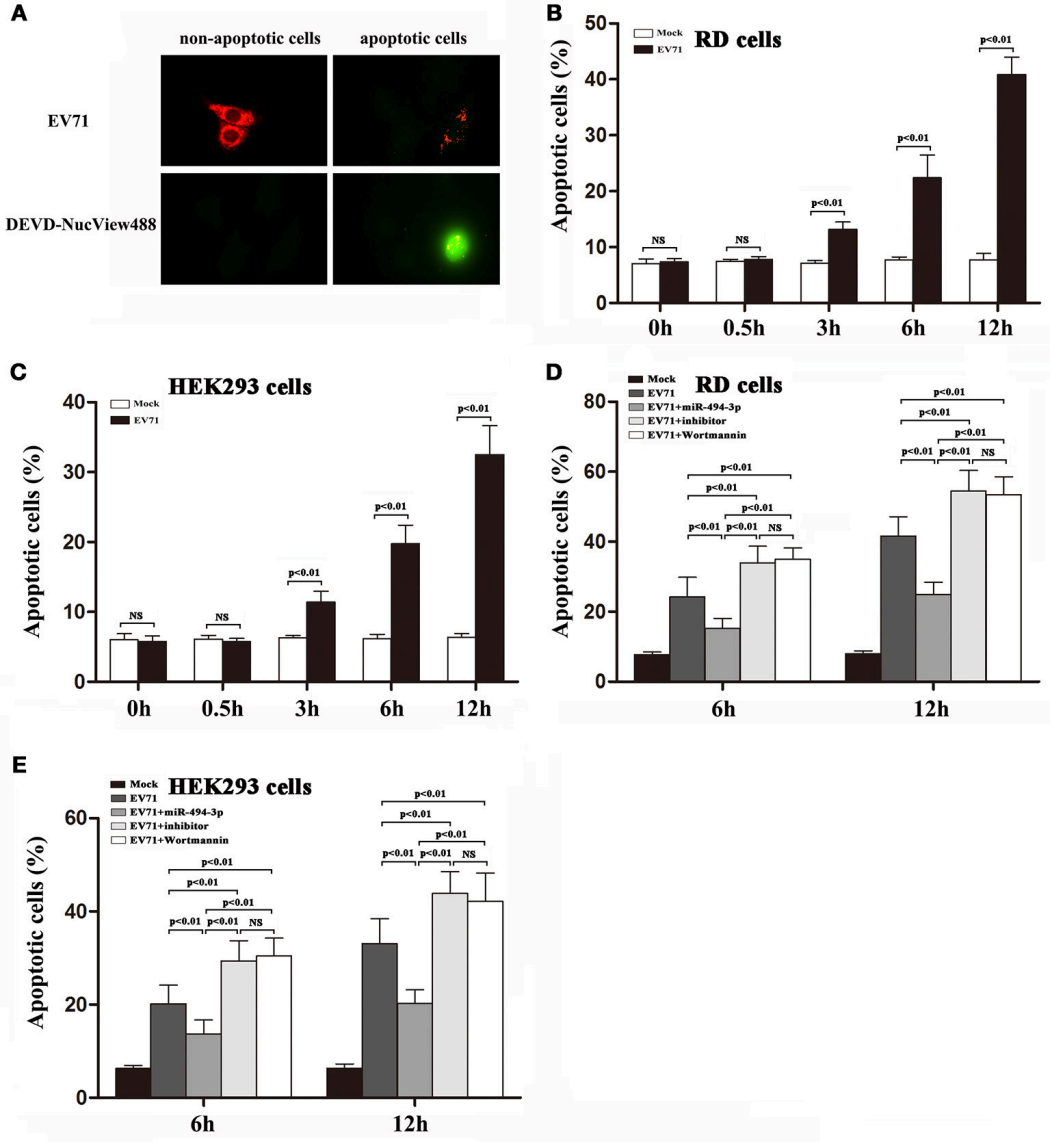
Activation of PI3K/Akt signaling pathway suppresses EV71-induced apoptosis in RD and HEK 293 cells. **(A)** RD cells were exposed to EV71 infection at an MOI of 2. After 12 h, the cells were pre-incubated for 30 min in the presence of NucView488 (5 μM). Fluorescence micrographs were taken at 12 hpi [representative images are shown; at least 5 individual cells were analyzed in each independent experiment (n = 3)]. **(B,C)** RD and HEK293 cells were infected with EV71 at an MOI of 2. At the indicated time points post-infection, the percentage of apoptotic cells was measured by Nucview 488 (Biotium) described in section Materials and Methods. **(D,E)** RD and HEK293 cells were transfected with or without hsa-miR-494-3p mimics or inhibitor for 48 h, or pre-incubated with or without Wortmannin (100 nM) for 1 h, and then followed by infection with EV71 for 6 and 12 h at an MOI of 2. Nucview 488 assay (flow cytometry) was employed to measure the percentage of apoptotic cells at the indicated time points. The experiments were repeated three times (*p* < 0.01 by ANOVA).

Over the years, several lines of evidence indicate that EV71 infection can lead to intrinsic cellular apoptosis, which in turn plays an important role in EV71 propagation (Wang et al., 2015). Finally, we employed NucView 488, a cell membrane-permeable fluorogenic caspase-3 substrate (Cen et al., 2008), to detect EV71-induced caspase-3 activity within the living cells in real time (Figure 7A). These studies indicate that (i) the EV71 infection could cause caspase-3 activation in both cell lines (Figures 7B,C), (ii) over-expression of hsa-miR-494-3p provided protection against EV71-induced cellular apoptosis, (iii) down-regulation of hsa-miR-494-3p via its inhibitor abolished protection against EV71-induced cell death, and (iv) a pharmacological inhibition of the PI3K/Akt signaling pathway with Wortmannin sensitized these cells to EV71-induced apoptosis (Figures 7D,E).

## Discussion

Over the past few decades, substantial evidence has been collected that EV71 infection depends on a wide variety of host factors, including miRNAs, receptors for EV71 entry, and cellular signaling pathways (Shih et al., 2011; Huang et al., 2012). Currently, the roles of miRNAs in EV71-host interactions have been paid more attention than before. This is perhaps best illustrated by the fact that the downregulation of the expression of hsa-miR-197 induced by EV71 infection promotes the expression of RAN, which facilitates the nuclear transport of viral proteins 3D/3CD and host protein hnRNP K (host heterogeneous nuclear ribonucleoprotein K) for viral propagation (Tang et al., 2015). Additionally, growing data indicate that the miRNA may serve as a double-edged sword in EV71 replication. It is well-known that hsa-miR-27a suppresses EV71 replication by regulating the *EGFR* (epidermal growth factor receptor) gene in infected cells (Zhang L. et al., 2014). On the other hand, it has been reported that the up-regulated expression of the cellular miRNA hsa-let-7c-5p induced by EV71 infection promotes viral replication by activating the JNK signaling pathway via targeting MAP4K4 (Zhou et al., 2017). However, it is important to recognize that no clinical trial has been performed to date to examine the feasibility of miRNAs in clinical practice among EV71 infected patients. Hence, more research needs to be done to investigate the molecular mechanisms underlying the roles of miRNAs in EV71 infection. In our study, we provide strong evidence that the increased hsa-miR-494-3p level is significantly higher in EV71-infected cells compared to the mock group. Importantly, we have shown that (i) the over-expression of hsa-miR-494-3p mimic level enhances EV71 propagation, and (ii) the inhibition of hsa-miR-494-3p levels via its inhibitor suppresses EV71 replication. Therefore, our data support the notion that hsa-miR-494-3p induced by EV71 infection may be involved in EV71 pathogenesis. In Supplementary Figure 1, we surprisingly found that UV-irradiated EV71 infection also can up-regulate the expression of hsa-miR-494-3p at 30 min pi. So far, the biological relevance of this process remains to be established. Future experiments need to be performed to get a better understanding of the effect of early-phase of hsa-miR-494-3p expression.

Until now, it is well-known that hsa-miR-494-3p, which is located on human chromosome 14q32.31, is involved in initiation, proliferation, invasion, and metastasis of numerous types of human cancers (Chen et al., 2015; Liu et al., 2015). However, the exact biological function of hsa-miR-494-3p in tumor cells remains controversial. For example, on one hand, it has been reported that hsa-miR-494-3p in human glioblastoma cells is up-regulated, which plays a key role in proliferation and migration of tumor cells via directly targeting PTEN, thereby resulting in the subsequent activation of the PI3K/Akt signaling pathway (Li et al., 2015). On the other hand, it has been shown that the activation of hsa-miR-494-3p exhibits a strong ability to reduce cancer stemness in head and neck squamous cell carcinomas by targeting Bmi1 and ADAM10, which correlates with longer survival time (Chang et al., 2015). For a long time, hsa-miR-494-3p was thought to function solely as oncogenic miRNA (Faversani et al., 2017). However, over the last years, hsa-miR-494-3p is increasingly recognized as a critical regulator in various physiological and pathological processes. This postulate is mainly based on these observations that (i) over-expression of hsa-miR-494-3p protects against ischemia/reperfusion-induced cardiac injury via targeting PTEN, ROCK1, and CaMKIIδ (Wang et al., 2010), and (ii) down-regulated hsa-miR-494-3p in the amygdale has been proven to be involved in the ethanol-induced anxiolysis (Teppen et al., 2016). Additionally, the interplay between hsa-miR-494-3p and pathogen has recently garnered attention due to the fact that the altered expression of hsa-miR-494-3p may be involved in the formation of chronic brucellosis (Budak et al., 2016). Remarkably, the results of two recent studies imply that EV71 infection can induce the up-regulation of hsa-miR-494-3p expression, though the authors did not explain the mechanism (Xun et al., 2015; Wang et al., 2016).

Currently, it is widely accepted that diverse signaling pathways are required for the EV71 replication (Peng et al., 2014). For example, our previous studies have already shown that MEK1-ERK signaling cascade acts as an important regulator of EV71 propagation (Wang et al., 2012). In addition, it has been reported that EV71 infection can induce the phosphorylation of MAPK/ERK and PI3K/Akt signaling, which in turn results in the phosphorylation and inactivation of GSK3β to delay cell apoptosis (Wong et al., 2005). And the same authors also provide evidence that the direct binding of EV71 to host cells could induce early phase of Akt phosphorylation. Importantly, there is growing evidence for the involvement of miRNAs-mediated signaling pathways in the etiology and progression of EV71-infected diseases. This assumption is mainly based on the observation that the knockdown of hsa-miR-876-5p, which is related to regulate the PI3K/Akt signaling pathway, results in a decline of EV71 viral RNA in neuroblastoma cells (Wang et al., 2016). Interestingly, our data also suggest that the EV71 infection triggers the activation of hsa-miR-494-3p-meidated PI3K/Akt signaling pathway, which plays a pivotal role in EV71 replication. Furthermore, we found that the PI3K/Akt signaling pathway induced by EV71 infection can subvert the anti-apoptotic pathway of the host cells. This finding extends a previous observation by Zhang et al. who reported that the activation of the PI3K/Akt signaling pathway limits the host cell apoptosis via inactivation of ASK1 during EV71 infection (Zhang H. et al., 2014).

So far, the relationship between the host cell apoptosis and EV71 replication remains unclear and controversial (Xi et al., 2013). Two potential mechanisms have been proposed. Concerning the first mechanism, there is some evidence that EV71 infection can induce intrinsic apoptosis in different types of cells, which leads to the initiation and progression of EV71-related disorders (Du et al., 2015; Wang et al., 2015; Xie et al., 2016). The second mechanism is mainly based on the observation that the EV71 infection can modulate the anti-apoptotic key regulators, GSK3β and ASK1, to delay cellular apoptosis, which in turn results in the enhancement of EV71 replication (Wong et al., 2005; Zhang H. et al., 2014). In this context, it is important to point out that we found that (i) hsa-miR-494-3p-induced by EV71 infection may target the PTEN, which activates the PI3K/Akt signaling pathway; and (ii) the activation of PI3K/Akt signaling pathway may be involved in the inhibition of early cellular apoptosis. Furthermore, inactivation of the PI3K/Akt pathway via hsa-miR-494-3p inhibitors or chemical inhibitor Wortmannin can significantly impact EV71 replication. Hence, these results imply that the up-regulation of hsa-miR-494-3 plays an important role in EV71 propagation. These findings confirm the previous observations suggesting that the PI3K/Akt signaling pathway acts as a crucial player in the regulation of viral protein expression and host cell apoptosis (Tung et al., 2010). However, although our study suggests that the hsa-miR-494-3p-PTEN-PI3K/Akt axis plays a pivotal role in EV71 replication, we cannot exclude the possibility that hsa-miR-494-3p may have other potential targets in host cells or viruses that could create a favorable environment for EV71 propagation.

In summary, our findings, combined with these previous reports, strongly indicate that hsa-miR-494-3p may be one of the key regulators that contributes to the initiation and development of EV71-infected diseases. This discovery opens up the way to potential new drug treatments that would combat EV71-related disorders. Importantly, further research with a large number of patients also needs to be done to investigate the molecular mechanisms underlying the role of hsa-miR-494-3p in EV71-related pathologies.

## Author contributions

W-XC and BW designed the study. QZ, YX, JX, SC, PL, YH, and YW produced and analyzed the data. All authors wrote the manuscript.

### Conflict of interest statement

The authors declare that the research was conducted in the absence of any commercial or financial relationships that could be construed as a potential conflict of interest.

## Supplementary material

Supplementary Figure 1 Ultraviolet-irradiated EV71 induces early-phase upregulation of hsa-miR-494-3p expression in RD and HEK 293 cells. The cells were exposed (open bars) or not exposed (closed bars) to UV-irradiated EV71 infection. The expression of hsa-miR-494-3p was quantified using qRCR at the indicated time points after EV71 infection. U6 was used as an internal control. The results represent the mean ± standard deviation of three independent experiments and present the relative expression levels of hsa-miR-494-3p in RD **(A)** and HEK 293 **(B)** cells (*p* < 0.01 by ANOVA).

Supplementary Figure 2 EV71 infection decreases the levels of PTEN via hsa-miR-494-3p. RD **(A)** and HEK293 **(B)** cells were transfected with or without hsa-miR-494-3p inhibitor for 48 h, and then followed by infection with EV71 for 12 h at an MOI of 2. At 12 h post-infection, the cells were collected and lysed for blotting analysis to detect PETN and β actin. β actin was used as an internal control. The experiments were repeated three times.

Supplementary Figure 3 Knockdown of PTEN enhances EV71 replication. RD **(A)** and HEK 293 **(B)** cells were transiently transfected with the siPTEN or siMut for 48 h. The cells were collected and lysed for blotting analysis to detect PETN and β actin. β actin was used as an internal control. After transfection with siRNA, the cells were infected with EV71 for 12 h at an MOI of 2. Cell lysates were blotted with anti-VP1 and anti-β actin antibodies. **(C,D)** Virus titers in the supernatants collected in panel **(E)** were measured by TCID_50_, and qRT-PCR was employed to determine the EV71 VP1 mRNA levels in RD and HEK 293 cells **(F)**. The data shown are the mean ± standard deviation. The experiments were repeated three times (*p* < 0.01 by ANOVA).

Supplementary Figure 4 Effect of hsa-miR-494-3p mimics and inhibitor on UV-EV71-mediated cell death. RD and HEK293 cells were transfected with or without hsa-miR-494-3p mimics or inhibitor for 48 h, and then followed by infection with UV-irradiated EV71 for 12 h. MTT assay was employed to evaluate the cell viability at the indicated time points. The experiments were repeated three times (*p* < 0.05, *p* < 0.01 by ANOVA).
