# Supplementary figures and images for: Host MicroRNA hsa-miR-494-3p Promotes EV71 Replication by Directly Targeting PTEN
Source: Front Cell Infect Microbiol. 2018 Sep 3;8:278. doi: 10.3389/fcimb.2018.00278 (PMC6130220; doi:10.3389/fcimb.2018.00278)

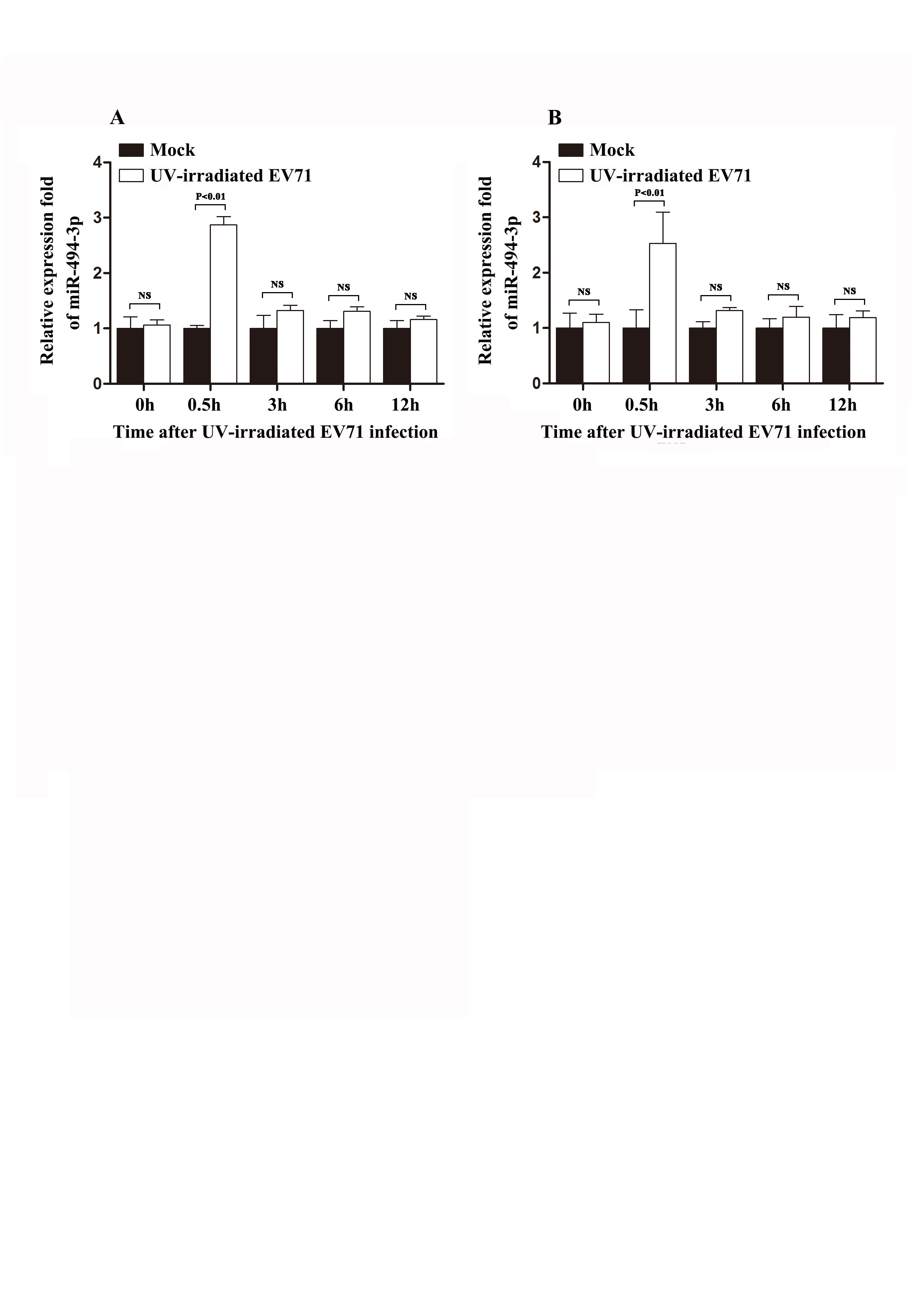

Supplement: Supplementary Figure 1 — Ultraviolet-irradiated EV71 induces early-phase upregulation of hsa-miR-494-3p expression in RD and HEK 293 cells. The cells were exposed (open bars) or not exposed (closed bars) to UV-irradiated EV71 infection. The expression of hsa-miR-494-3p was quantified using qRCR at the indicated time points after EV71 infection. U6 was used as an internal control. The results represent the mean ± standard deviation of three independent experiments and present the relative expression levels of hsa-miR-494-3p in RD (A) and HEK 293 (B) cells (p < 0.01 by ANOVA). [file Image_1.JPEG]

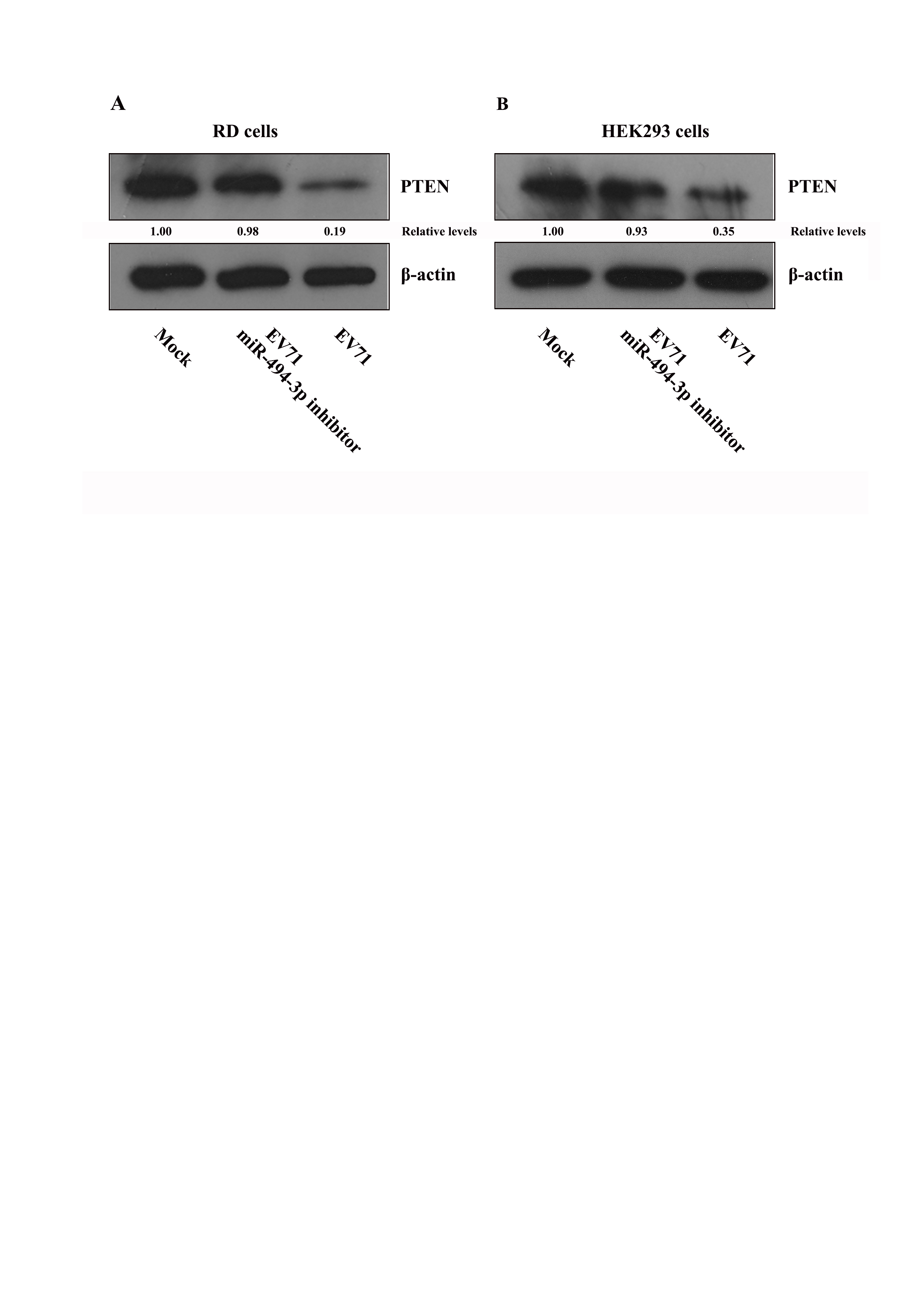

Supplement: Supplementary Figure 2 — EV71 infection decreases the levels of PTEN via hsa-miR-494-3p. RD (A) and HEK293 (B) cells were transfected with or without hsa-miR-494-3p inhibitor for 48 h, and then followed by infection with EV71 for 12 h at an MOI of 2. At 12 h post-infection, the cells were collected and lysed for blotting analysis to detect PETN and β actin. β actin was used as an internal control. The experiments were repeated three times. [file Image_2.JPEG]

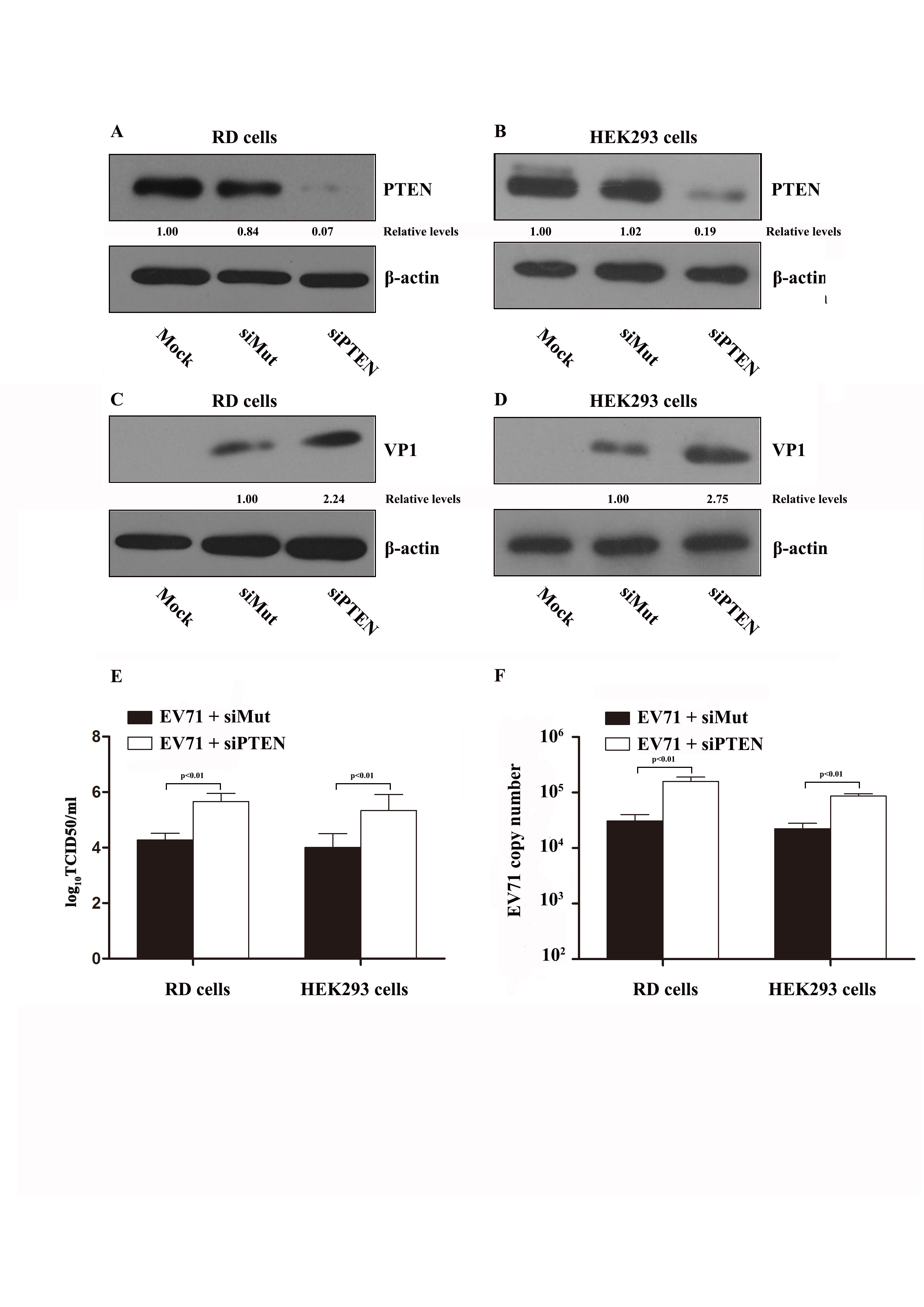

Supplement: Supplementary Figure 3 — Knockdown of PTEN enhances EV71 replication. RD (A) and HEK 293 (B) cells were transiently transfected with the siPTEN or siMut for 48 h. The cells were collected and lysed for blotting analysis to detect PETN and β actin. β actin was used as an internal control. After transfection with siRNA, the cells were infected with EV71 for 12 h at an MOI of 2. Cell lysates were blotted with anti-VP1 and anti-β actin antibodies. (C,D) Virus titers in the supernatants collected in panel (E) were measured by TCID50, and qRT-PCR was employed to determine the EV71 VP1 mRNA levels in RD and HEK 293 cells (F). The data shown are the mean ± standard deviation. The experiments were repeated three times (p < 0.01 by ANOVA). [file Image_3.JPEG]

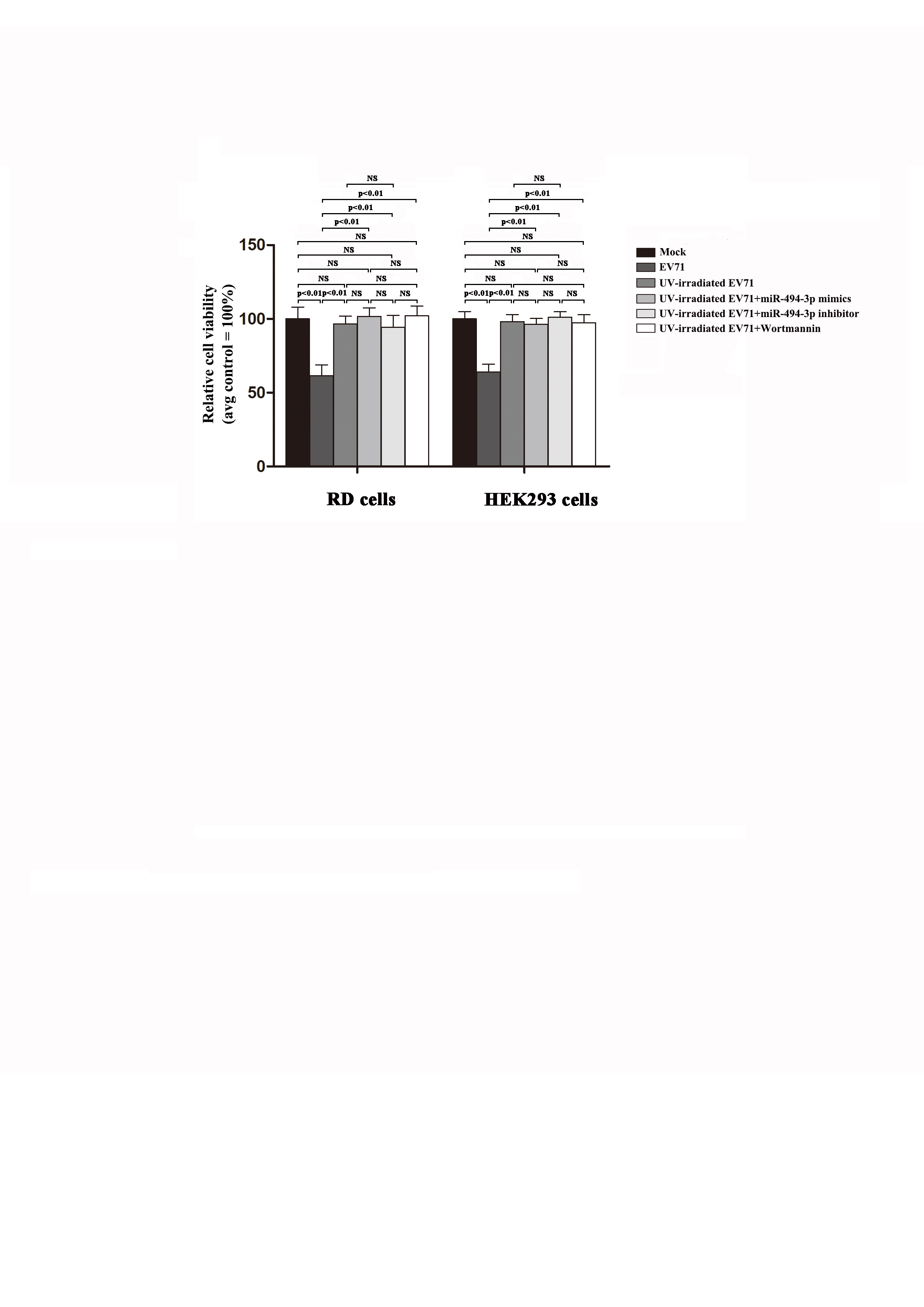

Supplement: Supplementary Figure 4 — Effect of hsa-miR-494-3p mimics and inhibitor on UV-EV71-mediated cell death. RD and HEK293 cells were transfected with or without hsa-miR-494-3p mimics or inhibitor for 48 h, and then followed by infection with UV-irradiated EV71 for 12 h. MTT assay was employed to evaluate the cell viability at the indicated time points. The experiments were repeated three times (p < 0.05, p < 0.01 by ANOVA). [file Image_4.JPEG]
